# *BRAF*, *PIK3CA*, and *HER2* Oncogenic Alterations According to *KRAS* Mutation Status in Advanced Colorectal Cancers with Distant Metastasis

**DOI:** 10.1371/journal.pone.0151865

**Published:** 2016-03-18

**Authors:** Soo Kyung Nam, Sumi Yun, Jiwon Koh, Yoonjin Kwak, An Na Seo, Kyoung Un Park, Duck-Woo Kim, Sung-Bum Kang, Woo Ho Kim, Hye Seung Lee

**Affiliations:** 1 Department of Pathology, Seoul National University Bundang Hospital, Seongnam, Republic of Korea; 2 Department of Pathology, Seoul National University College of Medicine, Seoul, Republic of Korea; 3 Department of Pathology, Kyungpook National University Hospital, Kyungpook National University School of Medicine, Daegu, Republic of Korea; 4 Department of Laboratory Medicine, Seoul National University Bundang Hospital, Seoul National University College of Medicine, Seongnam, Republic of Korea; 5 Department of Surgery, Seoul National University Bundang Hospital, Seongnam, Republic of Korea; Peter MacCallum Cancer Centre, AUSTRALIA

## Abstract

**Background:**

Anti-EGFR antibody–based treatment is an important therapeutic strategy for advanced colorectal cancer (CRC); despite this, several mutations—including *KRAS*, *BRAF*, and *PIK3CA* mutations, and *HER2* amplification—are associated with the mechanisms underlying the development of resistance to anti-EGFR therapy. The aim of our study was to investigate the frequencies and clinical implications of these genetic alterations in advanced CRC.

**Methods:**

*KRAS*, *BRAF*, and *PIK3CA* mutations were determined by Cobas real-time polymerase chain reaction (PCR) in 191 advanced CRC patients with distant metastasis. Microsatellite instability (MSI) status was determined by a fragmentation assay and *HER2* amplification was assessed by silver in situ hybridization. In addition, *KRAS* mutations were investigated by the Sanger sequencing method in 97 of 191 CRC cases.

**Results:**

Mutations in *KRAS*, *BRAF*, and *PIK3CA* were found in 104 (54.5%), 6 (3.1%), and 25 (13.1%) cases of advanced CRC, respectively. MSI-high status and *HER2* amplification were observed in 3 (1.6%) and 16 (8.4%) cases, respectively. *PIK3CA* mutations were more frequently found in *KRAS* mutant type (18.3%) than *KRAS* wild type (6.9%) (*P* = 0.020). In contrast, *HER2* amplifications and *BRAF* mutations were associated with *KRAS* wild type with borderline significance (*P* = 0.052 and 0.094, respectively). In combined analyses with *KRAS*, *BRAF* and *HER2* status, *BRAF* mutations or *HER2* amplifications were associated with the worst prognosis in the wild type *KRAS* group (*P* = 0.004). When comparing the efficacy of detection methods, the results of real time PCR analysis revealed 56 of 97 (57.7%) CRC cases with *KRAS* mutations, whereas Sanger sequencing revealed 49 cases (50.5%).

**Conclusions:**

*KRAS* mutations were found in 54.5% of advanced CRC patients. Our results support that subgrouping using *PIK3CA* and *BRAF* mutation or *HER2* amplification status, in addition to *KRAS* mutation status, is helpful for managing advanced CRC patients.

## Introduction

Colorectal cancer (CRC) is the third most common cancer and the incidence of CRC is still increasing worldwide annually. Despite of early detection and therapeutic advances, regional or distant metastatic disease accounts for almost 50% of newly diagnosed CRC patients and the overall survival rates of advanced CRC patients still remain unsatisfactory. The recent identification of molecular genetics has enabled considerable advancements in the management of patients with advanced CRC. The development of targeted therapies directed against specific mutations such as those in the *epidermal growth factor receptor (EGFR)* tyrosine kinase gene has improved treatment efficacy and clinical outcome in advanced CRC patients [1–4]. However, CRCs are molecularly heterogeneous tumors that harbor various gene alterations including mutations in *KRAS*, *BRAF*, and *PIK3CA*, as well as *HER2* amplification; many patients with these mutations therefore experience resistance against anti-EGFR drugs and exhibit poor prognosis [2,3,5,6]. Therefore, it is important to explore the molecular mechanism underlying the response and resistance to anti-EGFR treatment in advanced CRC.

*KRAS* mutations, which are commonly detected in approximately 40% of CRC cases, are thought to be associated with resistance to anti-EGFR treatment in CRC. The evaluation of *KRAS* mutations is thus essential prior to the use of anti-EGFR drugs to select patients who may benefit from anti-EGFR therapies [7,8]. Furthermore, recent studies suggest that additional gene mutations such as *BRAF* mutations, *PIK3CA* mutations, and *HER2* amplification are implicated in resistance to EGFR-targeted drugs for CRC patients with wild type *KRAS* [5,9]. *BRAF*, which is a member of RAF family, plays an important role in the MAP kinase/ERK-signaling pathway [10]. Many previous studies have revealed that mutations in *BRAF* are a biomarker for poor prognosis in advanced CRC. In addition, *BRAF* mutant tumors show a poor response to anti-EGFR treatment, especially in CRC patients with wild type *KRAS* [5,11]. *PIK3CA* is mutated in various human cancers; in CRC, it is mutated in approximately 20% of cases. Currently, patients harboring *PIK3CA* mutations in exon 20 and no mutations in *KRAS* may show resistance to anti-EGFR treatment. Moreover, *PIK3CA* mutations in exon 9 and KRAS mutations tend to be found together [3,12]. Finally, *HER2* amplifications are present in a small number of CRCs, and a few studies have reported the association between *HER2* amplification and poor response to anti-EGFR drugs [13].

Despite these previous findings, knowledge of the frequencies and clinical implications of these genetic alterations in Korean patients is still limited. In the present study, we evaluated the prevalence of these genetic alterations in patients with advanced CRC, and assessed the relationship of these genetic alterations with the clinicopathological factors and outcome of the patients. In addition, we compared the efficacy of using Cobas real-time polymerase chain reaction (PCR) tests with that of using Sanger sequencing tests as detection methods for *KRAS* mutations.

## Materials and Methods

### Patients and tissue samples

A total of 191 advanced CRC patients with synchronous or metachronous distant metastases who underwent surgical treatment at Seoul National University Bundang Hospital between 2003 and 2009 were enrolled in this study. All patients were treated with surgical resection of the primary CRCs at the initial diagnosis and distant metastasis resected when detected. None of the patients were treated with preoperative chemo- or radiotherapy. Clinicopathologic information and follow up data were obtained from the patients’ medical records and pathology reports. Overall survival (OS) was calculated as the time between the date of surgery and the date of death.

The histopathology and classification of the tumors were determined according to WHO classification. The use of medical record data and tissue samples for this study was approved by the Institutional Review Board of Seoul National University Bundang Hospital (reference: B-1210/174-301). All samples and medical record data were anonymized before use in this study and the participants did not provide written informed consent. The Institutional Review Board waived the need for written informed consent under the condition of anonymization and no additional intervention to the participants.

### *KRAS*, *BRAF*, and *PIK3CA* mutation analyses using the real-time PCR test

Tumor samples were collected from surgical resection specimens of the primary CRC. Hematoxylin-Eosin (HE) stained slides were reviewed by a pathologist (H.S.L). Tumor areas were identified and microscopically dissected more than a 1 x 1 cm area, which consisted of more than 60% tumor cells. One or two 8-μm-thick formalin-fixed paraffin-embedded (FFPE) tumor tissue sections were deparaffinized with xylene for 5 min at room temperature (RT), dehydrated in absolute alcohol for 5 min at RT, and allowed to air dry completely for 10 min. DNA was isolated using the Cobas DNA Sample Preparation Kit (Roche, Branchburg, NJ, USA) and the same preparation protocol for all Cobas mutation kits was used in this study. The concentration of the isolated DNA was measured using a NanoDrop UV spectrophotometer (Thermo Fisher Scientific, Wilmington, DE, USA) and the DNA was diluted with DNA Specimen Diluent from the Cobas 4800 Mutation Test kit (Roche) to the optimal concentration for each gene (*KRAS* 4 ng/μL, *BRAF* 5 ng/μL, and *PIK3CA* 2 ng/μL). Amplification and detection were performed with an Automated Cobas X480 analyzer instrument. The real-time PCR test could detect codon 12, 13, and 61 of *KRAS* mutation, V600E *BRAF* mutation, and exon 1, 4, 7, 9, and 20 of *PIK3CA* mutation.

### *KRAS* mutation analysis using the Sanger sequencing method

Tumor samples were collected from the same primary CRC specimens that had used for the real-time PCR tests. All specimens were microdissected manually and > 60% of the sample area was shown to contain tumor cells as estimated from the H&E-stained slides. Sanger sequencing analysis of *KRAS* mutations in codon 12, 13, and 61 was performed in 97 of the 191 FFPE tissue samples from CRC patients, as previously described [14].

### *HER2* analysis by dual-color silver in-situ hybridization (SISH)

*HER* analysis was performed on tissue array blocks from the same cohort. Construction of tissue array blocks was performed as previously described [5,15]. Briefly, a representative area of the 191 CRC case specimens was extracted, and two cores from central and peripheral area measuring 2 mm in diameter for each case was used for tissue array block construction. Bright-field dual-color SISH analysis was performed using an automatic SISH staining device (BenchMark XT, Ventana Medical Systems) according to the manufacturer’s protocols for the INFORM HER2 DNA and INFORM Chromosome 17 (CEP17) probes (Ventana Medical Systems). We interpreted HER2/CEP17 SISH signals according to the interpretive guide accompanying the INFORM HER2 DNA probe for staining gastric cancer cells (Ventana Medical systems). Tumor tissue was evaluated for hot spots of positive HER2/CEP17 signals using 20X or 40X objectives. Signals were enumerated in 20 non-overlapping tumor cell nuclei per core with 60X or 100X objectives. Small clusters were defined as 6 signals, and larger clusters as 12 signals. *HER2* gene amplification was defined as a HER2/CEP17 ratio of ≥ 2.0 in central or peripheral area. Those equivocal cases with a HER2/CEP17 ratio between 1.8 and 2.2 were recounted in 20 additional non-overlapping tumor cell nuclei; the ratio was recalculated based on these results.

### Microsatellite instability (MSI) analysis

Sections were prepared from FFPE tissue samples and hematoxylin and eosin–stained slides were evaluated to identify the representative tumor area and normal area in each section. These selected areas were microdissected. MSI analysis was performed as previously described [16,17]. Briefly, MSI status was determined by analyzing five microsatellite loci (BAT-26, BAT-25, D5S346, D17S250, and S2S123) using DNA auto-sequencer (ABI 3731 genetic analyzer; Applied Biosystems, Foster City, CA). According to the Bethesda guideline on MSI, tumors were classified as MSI-H when at least two of the five markers displayed novel bands, MSI-L when additional alleles were observed with one of the five markers, and MSS when all microsatellite markers examined displayed identical patterns in both tumor and normal tissues.

### Statistical analysis

Statistical analyses were performed with the SPSS Statistics 18 software package (Chicago, IL, USA). The association between the clinicopathologic parameters and genetic alterations were analyzed using the Chi-square test or Fisher’s exact test. The chi-square test was performed only if at least 80% of the cells have an expected frequency of 5 or greater, and no cell has an expected frequency smaller than 1.0. If not, Fisher’s exact test was used. Age was treated as a continuous variable and compared by using independent T test because of p>0.05 by Shapiro-Wilk normality test. Kaplan-Meier survival curves were plotted, and statistically significant differences in survival curves were analyzed using the log-rank test. Multivariate survival analysis using a Cox proportional hazards model was conducted with mutational status, age, and stage at initial diagnosis. The hazard ratio (HR) and its 95% confidence interval (CI) were evaluated. In all cases, *P* values less than 0.05 were considered statistically significant.

## Results

### Patient characteristics

The clinicopathologic features of the patients are summarized in S1 Table. Patients consisted of 103 men (53.9%) and 88 women (46.1%) with a median age of 60 years (range: 28–93 years). Of the 191 cases, 49 (25.7%) tumors were located in the right colon, 71 (37.2%) tumors in the left colon, and 71 (37.2%) tumors in the rectum. Regarding the histologic differentiation grade, 165 (86.4%) tumors were low grade, and 26 (13.6%) tumors were high grade. Regarding treatments, 176 (92.1%) patients received 5-fluorouracil (5-FU)–based adjuvant chemotherapy with or without anti-EGFR treatment (cetuximab) after surgical resection; 150 (85.2%) patients received 5-FU based chemotherapy only; and 26 (14.8%) patients received 5-FU with anti-EGFR drugs.

### Genetic alterations associated with EGFR signaling pathway in advanced CRCs

All the basic data are presented in S2 Table. Of the tumor cases examined, 87 (45.5%) had wild type *KRAS* and 104 (54.5%) had *KRAS* mutations. Among the tumors with *KRAS* mutations, mutations in codon 12 or 13 were observed in 97 (93.3%), whereas mutations in codon 61 were observed in 7 (6.7%) patients. *BRAF* (V600E) mutations were observed in 6 (3.1%) tumors. *PIK3CA* mutations were identified in 25 (13.1%) tumors. The two most common *PIK3CA* mutations were located in exon 9 (17 cases, 68.0%) and exon 20 (5 cases, 20.0%). Other rare mutations were located in exons 1 and 4 (2 cases, 8.0%). One case harbored a *PIK3CA* exon 4 mutation as well as *PIK3CA* exon 9 mutation. SISH analysis demonstrated *HER2* gene amplification in 16 (8.4%) tumors. Three cases from this cohort were MSI–H (1.6%), and the remaining 188 (98.4%) cases were classified as MSS/MSI-L.

Out of 104 *KRAS* mutant type CRC cases, 23 (22.1%) had *PIK3CA* mutations, *HER2* amplifications, or *BRAF* mutations (Table 1). Eighteen cases showed *PIK3CA* mutation, 4 cases showed *HER2* amplification, one case had both *BRAF* and *PIK3CA* mutations, and one case had both *PIK3CA* mutation and *HER2* amplification. Out of 87 *KRAS* wild type CRCs, *BRAF* mutations, *PIK3CA* mutations, and *HER2* amplifications were found in 5 (5.7%), 6 (6.9%), and 11 (12.6%) cases, respectively; overall, 21 of 87 *KRAS* wild type cases (24.1%) had *BRAF* mutations, *PIK3CA* mutations, or *HER2* amplifications.

**Table 1:** The frequencies of genetic alterations for the entire cohort of 191 advanced CRC patients.

Gene alteration	No.	%
***KRAS* mutation (n = 104)**		
*KRAS* only	81	42.4
*KRAS* and *PIK3CA*	17	8.9
*KRAS* and *HER2*	4	2.1
*KRAS*, *BRAF*, and *PIK3CA*	1	0.5
*KRAS*, *HER2*, and *PIK3CA*	1	0.5
***KRAS* wild type (n = 87)**		
All negative	66	34.6
*HER2* only	10	5.3
*PIK3CA* only	5	2.6
*BRAF* only	5	2.6
*HER2* and *PIK3CA*	1	0.5
Total	191	100

Interestingly, the presence of *PIK3CA* mutations was significantly associated with the presence of *KRAS* mutations (*P* = 0.020; Table 2). Mutations in *KRAS* and *BRAF* were nearly mutually exclusive; however, one case harbored concomitant *KRAS* and *BRAF* mutations. *HER2* amplifications and *BRAF* mutations tended to be more frequently observed in *KRAS* wild type tumors than in *KRAS* mutant type tumors with borderline statistical significance (*P* = 0.052 and *P* = 0.094, respectively). MSI status did not show any association with these genetic alterations in this cohort.

**Table 2:** Association of each genetic alteration.

Gene alteration	Total	*KRAS* wild type	*KRAS* mutant type	*P*
***BRAF* mutation**				0.094*
Wild type	185	82 (44.3%)	103 (55.7%)	
Mutant type	6	5 (83.3%)	1 (16.7%)	
***PIK3CA* mutation**				0.020
Wild type	166	81 (48.8%)	85 (51.2%)	
Mutant type	25	6 (24.0%)	19 (76.0%)	
***HER2* amplification**				0.052
Negative	175	76 (43.4%)	99 (56.6%)	
Positive	16	11 (68.8%)	5 (31.2%)	
**MSI status**				0.592*
MSS/MSI-L	188	85 (45.2%)	103 (54.8%)	
MSI-H	3	2 (66.7%)	1 (33.3%)	
**Total**	191	87 (45.5%)	104 (54.5%)	

KRAS, Kirsten rat sarcoma viral oncogene homolog; BRAF, v-raf murine sarcoma viral oncogene homolog B1; PIK3CA, phosphatidylinositol-4,5-bisphosphate 3-kinase catalytic subunit alpha; HER2, human epidermal growth factor receptor 2; MSI, microsatellite instability; MSS/MSI-L, microsatellite stable/MSI-low; MSI-H, MSI-high

*P-values are calculated by using Fisher’s exact test because less than 80% of the cells have an expected frequency of 5 or greater, or any cell has an expected frequency smaller than 1.0.

### Association of genetic alterations with clinicopathologic features

Table 3 demonstrates the relationship between genetic alterations and clinicopathologic characteristics. *KRAS* mutant tumors were more likely to be located in the right colon (*P* = 0.021). These tumors were also associated with low-grade histology (*P* = 0.029). *BRAF* mutant tumors were significantly associated with T4 depth of invasion (*P* = 0.033). Although it did not reach the statistical significance, *BRAF* mutant tumors tended to be located in the right colon (*P* = 0.127) and to have lymphatic invasion (*P* = 0.097) compared to the same features in *BRAF* wild type tumors. Tumors with *HER2* amplifications were significantly correlated with a distal location (*P* = 0.006). *HER2* amplifications also showed an association with younger, but this difference was not statistically significant (*P* = 0.081). There were no other significant associations between *PIK3CA* mutations or MSI status with clinicopathologic factors.

**Table 3:** Clinicopathologic characteristics according to mutational status of each gene.

		*KRAS* mutation		*BRAF* mutation		*PIK3CA* mutation		*HER2* amplification		MSI status	
Characteristic	Total	Mutant type	*P*	Mutant type	*P*	Mutant type	*P*	Positive	*P*	MSI-H	*P*
**Age**			0.503		0.676		0.347		0.081		0.131
Mean ± SD		60.32 ± 11.64		61.83 ± 14.69		61.92 ± 10.16		54.69 ± 11.31		70.33 ± 5.51	
**Sex**			0.752		0.688*		0.130		0.742		0.596*
Male	103	55 (53.4%)		4 (3.9%)		17 (16.5%)		8 (7.8%)		1 (1.0%)	
Female	88	49 (55.7%)		2 (2.3%)		8 (9.1%)		8 (9.1%)		2 (2.3%)	
**Location**			0.021		0.127*		0.282		0.006*		0.262*
Right	49	35 (71.4%)		4 (8.2%)		9 (18.4%)		2 (4.1%)		2 (4.1%)	
Left	71	35 (49.3%)		1 (1.4%)		10 (14.1%)		2 (2.8%)		1 (1.4%)	
Rectum	71	34 (47.9%)		1 (1.4%)		6 (8.5%)		12 (16.9%)		0 (0%)	
**Histologic grade**			0.029		0.189*		0.538*		0.242*		0.357*
Low	165	95 (57.6%)		4 (2.4%)		23 (13.9%)		12 (7.3%)		2 (1.2%)	
High	26	9 (34.6%)		2 (7.7%)		2 (7.7%)		4 (15.4%)		1 (3.8%)	
**T stage**			0.833		0.033*		0.563		0.520		0.561*
T1-T3	117	63 (53.8%)		1 (0.9%)		14 (12.0%)		11 (9.4%)		1 (0.9%)	
T4	74	41(55.4%)		5 (6.8%)		11 (14.9%)		5 (6.8%)		2 (2.7%)	
**pTNM stage**†			0.179*		1.000*		0.422*		0.306*		0.224*
I-	2	1 (50.0%)		1 (50%)		0 (0%)		0 (0%)		0 (0%)	
II	19	7 (52.9%)		0 (0%)		4 (21.1%)		1 (5.3%)		0 (0%)	
III	43	28 (65.1%)		1 (2.3%)		3 (7.0%)		1 (2.3%)		2 (4.7%)	
IV	127	68 (53.5%)		5 (3.9%)		18 (14.2%)		14 (11.0%)		1 (0.8%)	
**Lymphatic invasion**			0.622		0.097*		0.499		0.806		1.000*
Absent	65	37 (56.9%)		0 (0%)		10 (15.4%)		5 (7.7%)		1 (1.5%)	
Present	126	67 (53.2%)		6 (4.8%)		15 (11.9%)		11 (8.7%)		2 (1.6%)	
**Venous invasion**			0.148		1.000*		0.458		0.780*		1.000*
Absent	133	77 (57.9%)		4 (3.0%)		19 (14.3%)		12 (9.0%)		2 (1.5%)	
Present	58	27 (46.6%)		2 (3.4%)		6 (10.3%)		4 (6.9%)		1 (1.7%)	
**Perineural invasion**			0.896		0.685*		0.640		0.844		1.000*
Absent	91	50 (54.9%)		2 (2.2%)		13 (14.3%)		8 (8.8%)		1 (1.1%)	
Present	100	54 (54.0%)		4 (4.0%)		12 (12.0%)		8 (8.0%)		2 (2.0%)	

KRAS, Kirsten rat sarcoma viral oncogene homolog; BRAF, v-raf murine sarcoma viral oncogene homolog B1; PIK3CA, phosphatidylinositol-4,5-bisphosphate 3-kinase catalytic subunit alpha; HER2, human epidermal growth factor receptor 2; MSS, microsatellite stable; MSI-L, microsatellite instability-low; MSI-H, microsatellite instability-high; SD, standard deviation

Age was compared between two groups by using independent T test.

*P-values are calculated by using Fisher’s exact test because less than 80% of the cells have an expected frequency of 5 or greater, or any cell has an expected frequency smaller than 1.0.

†Stage is the stage at initial diagnosis.

### Prognostic significance of genetic alterations

**Fig 1:**
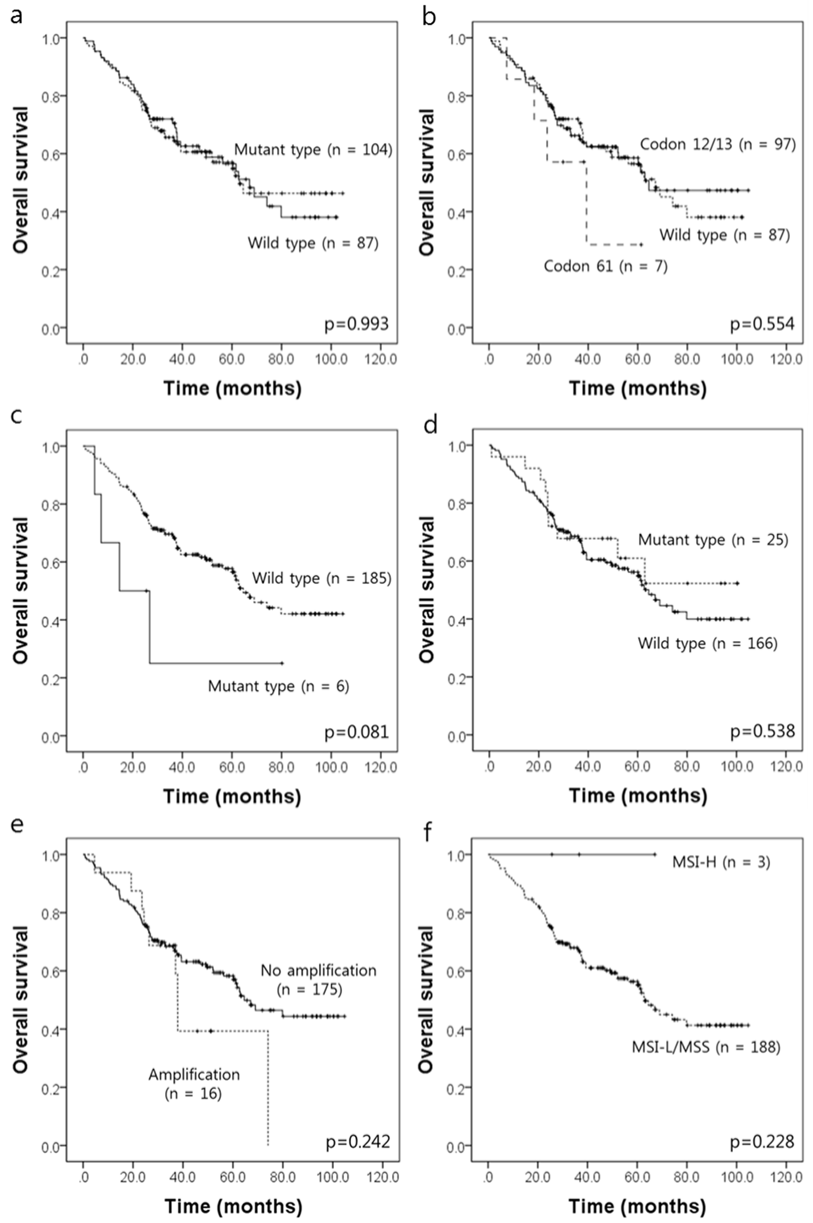
Kaplan-Meier survival estimate graphs of overall survival (OS) in 191 advanced CRC patients according to *KRAS* mutations status (a), locations of *KRAS* mutations in advanced CRC patients (b), *BRAF* mutations (c), *PIK3CA* mutations (d), *HER2* amplifications (e), and MSI status (f).

To determine the prognostic significance of these genetic alterations, survival analyses were performed using the Kaplan-Meier method for OS (Fig 1). Follow up data from all 191 CRC patients were included in the survival analysis. There were 84 CRC-related deaths, and the median follow up time was 37.9 months (range, 0.8–104.6 months). Patients with *BRAF* mutations showed a tendency for unfavorable outcome for OS, but this result did not reach statistical significance (*P* = 0.081). *KRAS* mutations, *PIK3CA* mutations, *HER2* amplifications, and MSI status did not show any association with the patients’ OS (*P* = 0.993, *P* = 0.538, *P* = 0.368, and *P* = 0.538, respectively). Mutation of *KRAS* codon 61 tended to be associated with shorter overall survival, but it did not reach statistical significance (*P* = 0.554).

**Fig 2:**
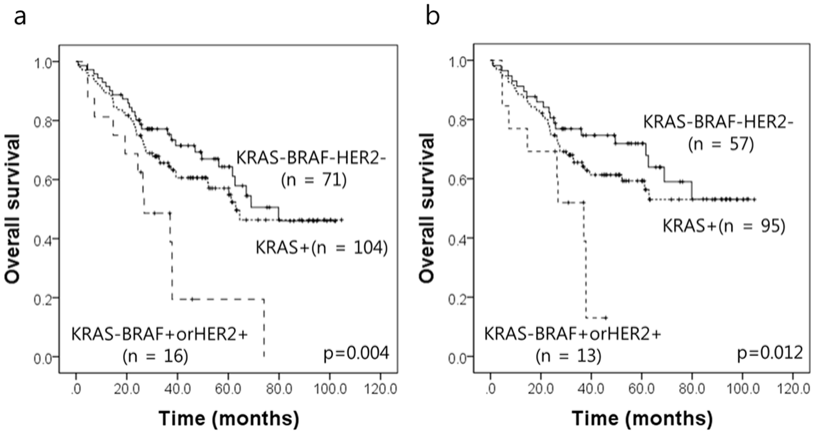
Results of combined analysis in advanced CRC patients with the *KRAS* wild type subgroup according to *BRAF* mutation and *HER2* amplification regardless of anti-EGFR treatment status (n = 191) (a), in whom were not treated with anti-EGFR drugs (n = 165) (b).

Interestingly, the *KRAS* wild type subgroup with *BRAF* mutations or *HER2* amplifications showed the worst prognosis in combined analyses (*P* = 0.004; Fig 2A). By using Cox proportional hazards model, this subgroup was poor prognostic factor (HR, 2.055; CI, 1.093–3.861; *P* = 0.025) independently of age and stage at initial diagnosis (S3 Table). Among 191 advanced CRCs, 165 patients were not treated with anti-EGFR drugs patients, in whom the *KRAS* wild type subgroup with *BRAF* or *HER2* alterations also showed the worst prognosis (*P* = 0.012; Fig 2B). By using Cox proportional hazards model, this subgroup was poor prognostic factor with borderline statistical significance (HR, 1.984; CI, 0.963–4.085; *P* = 0.063; data not shown). However, in 26 patients treated with 5-FU with anti-EGFR drugs, the *KRAS* wild type subgroup with *BRAF* or *HER2* alterations was not associated with poor prognosis (*P* = 0.305, data not shown), which may be because of small number of cases. In the *KRAS* wild type subgroup, *BRAF* or *HER2* alterations was associated with high grade histologic differentiation, advanced stage, lymphatic invasion and perineural invasion, but with borderline statistical significance (S4 Table).

### Comparison of Cobas real-time PCR and Sanger sequencing methods for *KRAS* mutations

Of 191 CRC samples, the tissues of 97 patients were available for analysis to compare the detection of *KRAS* mutations with the Sanger sequencing test and Cobas real-time PCR test. Of the 97 tumors included, *KRAS* mutations were detected in 49 cases (50.5%) by the Sanger sequencing test. Mutations in *KRAS* codon 12 or 13 and *KRAS* codon 61 were detected in 47 (48.5%) and 2 (2.1%) cases, respectively. On the other hand, 56 cases (57.7%) of *KRAS* mutations were detected by the real-time PCR test; the test located 52 (53.6%) mutations in codon 12 or 13 and 4 (4.1%) mutations in codon 61. The real-time PCR test showed a higher sensitivity than that of the Sanger sequencing test.

## Discussion

*KRAS* is a well-known driver oncogene in CRCs and the presence of *KRAS* mutation predicts poor response to anti-EGFR targeted therapy in metastatic CRC patients. We evaluated the frequencies and clincopathologic significance of mutations in *KRAS*, *BRAF*, and *PIK3CA*, and *HER2* amplification, as well as the relationship of these genetic alterations in advanced CRC patients who were candidates for anti-EGFR treatment in daily practice. Mutations in *KRAS*, *BRAF*, and *PIK3CA* were found in 104 (54.5%), 6 (3.1%), and 25 (13.1%) cases of advanced CRC, respectively. In addition, MSI-H phenotype and *HER2* amplification were observed in 3 (1.6%) and 16 (8.4%) cases, respectively.

The development of targeted therapies against specific molecular alterations has contributed to the management of advanced CRC patients, and anti-EGFR drugs are used in these patients. Although the presence of *KRAS* mutation is useful to exclude patients who will not benefit from anti-EGFR treatment, many patients with wild type *KRAS* CRC show negative responses to this treatment. To date, it is considered that *BRAF* mutations, *PIK3CA* mutations, and *HER2* amplifications are associated with the underlying mechanisms of these poor responses [4,5]. In our cohort, 16 of 87 *KRAS* wild type cases (18.4%) had *BRAF* mutations or *HER2* amplifications; furthermore, combined analysis showed that *KRAS* wild type patients with *BRAF* mutations or *HER2* amplifications had the worst prognosis. Because the targeted therapies to *BRAF* mutations and *HER2* amplifications have significant survival benefit in various human cancers [10,18,19], treatment with anti-BRAF and anti-HER2 agents may be a good therapeutic strategy to improve survival in CRC patients with wild type *KRAS* harboring *BRAF* mutations or *HER2* amplifications and who also have primary or secondary resistance to anti-EGFR treatment.

Despite a small number of tumors with *PIK3CA* mutations in our cohort, we found that *PIK3CA* mutations largely overlapped with *KRAS* mutations, which was consistent with the previous studies in European [20] and Japanese [21] CRC patients with metastasis. Currently, several inhibitors targeting the *PIK3CA* signaling pathway have been developed, and these agents are being tested in preclinical and clinical trials of patients with CRC [22–24]. Considering our result and the previous studies [20,21] that *KRAS* mutations often coexisted with *PIK3CA* mutations, inhibiting the *PIK3CA* signaling pathway might be a useful therapeutic strategy to treat CRC patients with *KRAS* mutations.

Overall, 24 of 191 cases (12.6%) had two or more oncogenic alterations in this study. It may be interpreted that these genetic alterations occur at the same time in the same tumor, but it may be due to tumor heterogeneity. During tumor progression, oncogenic alterations develop in a subclone, which contribute to cancer metastasis and drug resistance. We used more sensitive detection methods, thus mutations in minor tumor cell population could be detected. In managing CRC patients, sensitive molecular diagnosis is helpful to detect minor oncogenic alterations, which can be the next target in advanced CRC patients with primary or secondary resistance to the first-line targeted treatment. In our cohort, one case harbored concomitant *KRAS* and *BRAF* mutations. It is well known that *BRAF* mutations are usually detected in *KRAS* wild type tumors, and that they are almost mutually exclusive with *KRAS* mutations in CRC. Several studies have reported that rare cases harbor combined *KRAS* and *BRAF* mutations, which occurs in less than 0.02% [25,26]. Even though the tumor biology and prognosis of patients with concomitant *KRAS* and *BRAF* mutations have been still uncertain, previous research suggests that these concomitant mutations are associated with tumor progression such as lymph node metastasis and higher T stage [25,27]. Further large-scale studies are needed to clarify the incidence and biologic function of concomitant *KRAS* and *BRAF* mutations.

Previous studies with advanced CRC patients with metastasis reported that approximately 34~45% of the patients had *KRAS* mutations [20,21,28]. This study with Korean CRC patients demonstrated that the frequency of *KRAS* mutations was 54.5%, and that these mutations were seen mainly in codons 12 or 13 (93.3%). The frequency of *KRAS* mutations in this cohort was slightly higher than that in previously published reports of metastatic CRC patients [20,21,28]. There are several possible explanations for it. First, we enrolled only advanced CRC patients in this cohort, which may account for the higher frequency of *KRAS* mutations. Second, there were some differences in mutation detection methods. We analyzed mutation status using Cobas real-time PCR, which is considered to show higher sensitivity than that of other detection methods.

It has been reported that most of *KRAS* mutations in CRC patients occur in codon 12 and 13. This study also demonstrated that *KRAS* mutations were seen mainly in codons 12 or 13 (93.3%). In our *KRAS* mutation subgroup analysis, mutation of *KRAS* codon 61 tended to be associated with shorter overall survival, but it did not reach statistical significance. The prognostic impact of *KRAS* codon 61 mutations has been reported in several previous studies, though with controversial results [29]. Because the incidence of *KRAS* codon 61 mutations is rare, further large-scale studies may help clarify the relationship between these mutations and clinical outcome.

Mutations of *BRAF* and *PIK3CA* were detected in 3.1% and 13.1% of cases, respectively. Although the frequencies of these mutations were considered to be low, they were similar to those of previous studies in advanced CRC [20,21,28]. In agreement with previous study [30], *BRAF* mutations were associated with aggressive CRC histology, such as higher T stage and presence of lymphatic invasion. Though the results did not reach statistical significance, patients harboring *BRAF* mutations also showed shorter OS.

We evaluated two detection methods for *KRAS* mutations in CRC samples: Cobas real-time PCR and Sanger sequencing. There was a good correlation between *KRAS* mutation detection by real-time PCR and that of Sanger sequencing, and real-time PCR showed higher sensitivity than that of Sanger sequencing. In our cohort, 7 cases of *KRAS* mutations were detected by the real-time test that were not detected by the Sanger sequencing test, specifically 5 cases for codon 12 or 13 and 2 cases for codon 61. The Sanger sequencing method, developed in 1975, was considered one of the basic mutation detection methods; however, this method appears to have limited sensitivity—a low level of the mutant allele may be undetectable by this method [31]. Conversely, the real-time PCR test including various commercial kits is considered to be a highly sensitive method that shows advantages in detecting *KRAS* mutations [32]. To date, the considerable intratumoral heterogeneity of molecular alterations and their clinical impact on the targeted therapy have been described in various tumors. In CRC, several studies reported the clinical significance of *KRAS* heterogeneity in anti-EGFR treatment [5,33,34]. Normanno et al. suggested that a low content of *KRAS* mutant alleles was sufficient to produce resistance to EGFR monoclonal antibodies, and the threshold in their study was 3~10% of mutant *KRAS* allele frequency which was unlikely to be detected by using Sanger sequencing [33]. Although further studies are needed to validate these results and clarify the role of the molecular alterations to resistance to anti EGFR treatment, the accurate detection of these mutations has great clinical significance.

In conclusion, we found that the prevalence of *KRAS* mutations was 54.5% in Korean advanced CRC patients, which was more frequent than that reported in other populations. *BRAF* mutations or *HER2* amplifications were found in 16.1% of *KRAS* wild type patients, and furthermore, combined analysis showed that *KRAS* wild type patients with *BRAF* or *HER2* amplifications had the worst prognosis. *PIK3CA* mutations were more frequently observed in *KRAS* mutant type than in wild type *KRAS* CRC patients. Therefore, subgrouping depending on the status of *PIK3CA* and *BRAF* mutation or *HER2* amplification, in addition to *KRAS* mutation status, is helpful to determine CRC patient management strategies. We also demonstrated that a real time PCR method had high sensitivity for detecting *KRAS* mutations in CRC patients.

## Supporting Information

S1 Table Clinicopathologic characteristics of 191 advanced CRC patients. (DOCX)

S2 Table The basic clinicopathologic findings of each patient. (DOCX)

S3 Table Multivariate analysis for factors predictive of survival (Cox proportional hazards model). (DOCX)

S4 Table Association between clinicopathologic characteristics and *BRAF* or *HER2* alterations in *KRAS* wild type CRCs. (DOCX)
