# Supplementary material for: BRAF, PIK3CA, and HER2 Oncogenic Alterations According to KRAS Mutation Status in Advanced Colorectal Cancers with Distant Metastasis
Source: PLoS One. 2016 Mar 18;11(3):e0151865. doi: 10.1371/journal.pone.0151865 (PMC4798471; doi:10.1371/journal.pone.0151865)
Supplement: S1 Table — (DOCX) [file pone.0151865.s001.docx]

**S1 Table.** Clinicopathologic characteristics of 191 advanced CRC patients

| Characteristic | No. | % |
| --- | --- | --- |
| **Age** |  |  |
| Mean (standard deviation) | 59.77 | 12.20 |
| **Gender** |  |  |
| Male | 103 | 53.9 |
| Female | 88 | 46.1 |
| **Location** |  |  |
| Right | 49 | 25.7 |
| Left | 71 | 37.2 |
| Rectum | 71 | 37.2 |
| **T-stage** |  |  |
| T1-T3 | 117 | 61.3 |
| T4 | 74 | 38.7 |
| **Histologic grade** |  |  |
| Low Grade | 165 | 86.4 |
| High Grade | 26 | 13.6 |
| **Stage*** |  |  |
| I | 2 | 1.0 |
| II | 19 | 9.9 |
| III | 43 | 22.5 |
| IV | 127 | 66.5 |
| **LN metastasis** |  |  |
| Present | 156 | 81.7 |
| Absent | 35 | 18.3 |
| ***KRAS* mutation** |  |  |
| Wild type | 87 | 45.5 |
| Mutant type | 104 | 54.5 |
| (codon 12/13) | (97) | (50.8) |
| (codon 61) | (7) | (3.7) |
| ***BRAF* mutation (V600E)** |  |  |
| Wild type | 185 | 96.9 |
| Mutant type | 6 | 3.1 |
| ***PIK3CA* mutation** |  |  |
| Wild type | 166 | 86.9 |
| Mutant type | 25 | 13.1 |
| ***HER2* amplification** |  |  |
| Negative | 175 | 91.6 |
| Positive | 16 | 8.4 |
| **MSI** |  |  |
| MSS/MSI-L | 188 | 98.4 |
| MSI-H | 3 | 1.6 |

CRC, colorectal cancer; LN, lymph node; KRAS, Kirsten rat sarcoma viral oncogene homolog; BRAF, v-raf murine sarcoma viral oncogene homolog B1; PIK3CA, phosphatidylinositol-4,5-bisphosphate 3-kinase catalytic subunit alpha; HER2, human epidermal growth factor receptor 2

*Stage is the stage at initial diagnosis.
