# Supplementary material for: BRAF, PIK3CA, and HER2 Oncogenic Alterations According to KRAS Mutation Status in Advanced Colorectal Cancers with Distant Metastasis
Source: PLoS One. 2016 Mar 18;11(3):e0151865. doi: 10.1371/journal.pone.0151865 (PMC4798471; doi:10.1371/journal.pone.0151865)
Supplement: S2 Table — (DOCX) [file pone.0151865.s002.docx]

**S2 Table.** The basic clinicopathologic findings of each patient

|  | age | Gender | Location | pT stage | differentiation | LN metastasis | Stage* | survival | FU months | *KRAS^§^* | *BRAF* | *PIK3CA* | *HER2* | MSI |
| --- | --- | --- | --- | --- | --- | --- | --- | --- | --- | --- | --- | --- | --- | --- |
| Case 1 | 75 | male | left | 3 | LG | present | 3 | alive | 104.6 | Codon12/13 | ND | ND | N | MSS |
| Case 2 | 64 | female | left | 4 | LG | present | 4 | alive | 17.7 | ND | ND | ND | N | MSS |
| Case 3 | 63 | male | left | 3 | LG | present | 4 | dead | 79.8 | ND | ND | ND | N | MSS |
| Case 4 | 40 | male | left | 4 | LG | present | 3 | dead | 64.5 | Codon12/13 | ND | ND | N | MSS |
| Case 5 | 64 | male | left | 3 | HG | absent | 2 | alive | 102.3 | ND | ND | ND | N | MSS |
| Case 6 | 51 | female | rectum | 3 | LG | absent | 2 | alive | 101.8 | ND | ND | ND | N | MSS |
| Case 7 | 57 | female | rectum | 3 | LG | absent | 4 | dead | 52.2 | Codon12/13 | ND | ND | N | MSS |
| Case 8 | 51 | male | rectum | 4 | LG | present | 4 | dead | 26.5 | Codon12/13 | ND | ND | N | MSS |
| Case 9 | 58 | male | rectum | 3 | LG | absent | 2 | alive | 100.4 | Codon12/13 | ND | E542K | N | MSS |
| Case 10 | 66 | male | rectum | 3 | LG | absent | 2 | alive | 100.2 | Codon12/13 | ND | E545X^†^, R88Q | N | MSS |
| Case 11 | 35 | female | rectum | 3 | LG | present | 3 | dead | 25.1 | ND | ND | ND | N | MSS |
| Case 12 | 62 | male | rectum | 3 | LG | absent | 2 | alive | 98.8 | ND | ND | ND | N | MSS |
| Case 13 | 76 | female | left | 3 | HG | absent | 2 | dead | 36.1 | ND | ND | ND | N | MSS |
| Case 14 | 60 | female | left | 4 | LG | absent | 4 | alive | 98 | Codon12/13 | ND | ND | N | MSS |
| Case 15 | 66 | male | left | 3 | LG | absent | 2 | alive | 97.7 | Codon12/13 | ND | ND | N | MSS |
| Case 16 | 43 | female | right | 3 | LG | present | 4 | dead | 61.1 | Codon12/13 | ND | ND | N | MSS |
| Case 17 | 93 | female | right | 4 | LG | absent | 4 | dead | 21.7 | Codon12/13 | ND | ND | N | MSS |
| Case 18 | 56 | male | right | 4 | LG | present | 4 | alive | 95.3 | Codon12/13 | ND | E542K | N | MSS |
| Case 19 | 39 | male | rectum | 2 | LG | absent | 1 | alive | 94.9 | ND | ND | ND | N | MSS |
| Case 20 | 28 | female | left | 4 | HG | present | 3 | dead | 67.1 | ND | ND | ND | N | MSS |
| Case 21 | 60 | male | right | 4 | LG | present | 4 | dead | 52 | Codon12/13 | ND | H1047X^†^ | N | MSS |
| Case 22 | 38 | male | rectum | 3 | HG | present | 4 | dead | 74.1 | ND | ND | ND | AMP | MSS |
| Case 23 | 73 | female | rectum | 4 | LG | present | 3 | alive | 93.7 | ND | ND | E542K | N | MSS |
| Case 24 | 57 | female | right | 3 | HG | present | 4 | alive | 93.4 | Codon12/13 | ND | ND | N | MSS |
| Case 25 | 44 | male | rectum | 3 | LG | absent | 2 | alive | 93.4 | ND | ND | ND | N | MSS |
| Case 26 | 65 | male | right | 3 | LG | present | 4 | dead | 60.2 | Codon12/13 | ND | ND | N | MSS |
| Case 27 | 45 | female | left | 4 | LG | present | 4 | alive | 92 | Codon12/13 | ND | ND | N | MSS |
| Case 28 | 51 | female | rectum | 3 | LG | present | 4 | dead | 56.2 | ND | ND | ND | N | MSS |
| Case 29 | 63 | female | rectum | 3 | LG | present | 4 | alive | 91.5 | ND | ND | ND | N | MSS |
| Case 30 | 44 | female | left | 4 | HG | present | 4 | dead | 10.4 | ND | ND | ND | N | MSS |
| Case 31 | 55 | male | rectum | 3 | HG | present | 4 | dead | 7 | ND | ND | ND | N | MSS |
| Case 32 | 73 | female | right | 4 | LG | present | 4 | dead | 13.1 | Codon12/13 | ND | ND | N | MSS |
| Case 33 | 36 | male | left | 3 | LG | absent | 2 | alive | 89.3 | ND | ND | ND | N | MSS |
| Case 34 | 59 | male | rectum | 3 | LG | present | 3 | alive | 88.9 | Codon12/13 | ND | ND | N | MSS |
| Case 35 | 85 | female | left | 4 | LG | present | 4 | dead | 0.9 | ND | ND | E542K | N | MSS |
| Case 36 | 82 | male | right | 3 | HG | present | 4 | dead | 4.2 | ND | ND | ND | N | MSS |
| Case 37 | 48 | female | rectum | 3 | LG | absent | 2 | alive | 88 | Codon12/13 | ND | ND | N | MSS |
| Case 38 | 41 | female | right | 4 | LG | absent | 4 | dead | 36.6 | Codon12/13 | ND | ND | N | MSS |
| Case 39 | 68 | female | rectum | 3 | LG | present | 3 | dead | 33 | Codon12/13 | ND | ND | N | MSS |
| Case 40 | 70 | male | left | 3 | LG | absent | 2 | alive | 85.8 | ND | ND | ND | N | MSS |
| Case 41 | 54 | male | left | 3 | LG | present | 4 | dead | 39.4 | Codon61 | ND | ND | N | MSS |
| Case 42 | 73 | female | left | 4 | LG | present | 4 | dead | 1.4 | Codon12/13 | ND | ND | N | MSS |
| Case 43 | 82 | male | right | 4 | HG | present | 4 | dead | 7.1 | Codon61 | ND | ND | N | MSS |
| Case 44 | 58 | male | rectum | 2 | LG | absent | 1 | alive | 71.8 | Codon12/13 | ND | ND | N | MSS |
| Case 45 | 70 | female | right | 4 | LG | absent | 4 | dead | 14.8 | Codon12/13 | ND | ND | N | MSS |
| Case 46 | 64 | male | left | 4 | HG | present | 4 | dead | 20.5 | ND | ND | ND | N | MSS |
| Case 47 | 56 | female | right | 4 | HG | present | 3 | dead | 13.3 | ND | ND | ND | N | MSS |
| Case 48 | 77 | female | right | 4 | LG | present | 4 | dead | 2.4 | Codon12/13 | ND | ND | N | MSS |
| Case 49 | 67 | female | right | 4 | LG | present | 3 | dead | 39.4 | Codon12/13 | ND | ND | N | MSS |
| Case 50 | 58 | male | rectum | 4 | LG | present | 4 | dead | 26.9 | Codon12/13 | ND | ND | N | MSS |
| Case 51 | 72 | male | rectum | 3 | LG | present | 4 | dead | 14.7 | Codon12/13 | ND | ND | N | MSS |
| Case 52 | 62 | male | right | 4 | LG | present | 4 | dead | 22.8 | Codon12/13 | ND | Q546X^†^ | N | MSS |
| Case 53 | 51 | female | rectum | 3 | LG | present | 4 | alive | 67.6 | ND | ND | ND | N | MSS |
| Case 54 | 67 | male | right | 3 | LG | present | 3 | alive | 67.1 | Codon12/13 | ND | ND | N | MSS |
| Case 55 | 74 | female | right | 4 | HG | present | 3 | alive | 67.1 | ND | ND | ND | N | MSI-H |
| Case 56 | 47 | female | right | 3 | LG | present | 3 | dead | 25.8 | Codon12/13 | ND | ND | N | MSS |
| Case 57 | 38 | male | rectum | 3 | LG | present | 4 | alive | 65.6 | ND | ND | ND | N | MSS |
| Case 58 | 66 | female | rectum | 4 | LG | present | 4 | dead | 4.6 | ND | ND | ND | AMP | MSS |
| Case 59 | 38 | female | rectum | 3 | LG | present | 4 | dead | 37.8 | ND | ND | ND | AMP | MSS |
| Case 60 | 62 | male | rectum | 3 | LG | present | 4 | dead | 32.8 | Codon12/13 | ND | ND | N | MSS |
| Case 61 | 56 | male | left | 3 | LG | present | 3 | alive | 63.4 | Codon12/13 | ND | ND | N | MSS |
| Case 62 | 58 | female | right | 4 | LG | present | 4 | alive | 63.4 | Codon12/13 | ND | E545X^†^ | N | MSS |
| Case 63 | 71 | male | left | 4 | LG | present | 4 | dead | 46.9 | ND | ND | ND | N | MSS |
| Case 64 | 60 | female | right | 3 | LG | absent | 3 | alive | 63.1 | Codon12/13 | ND | ND | N | MSS |
| Case 65 | 59 | female | left | 3 | LG | present | 3 | alive | 61.7 | Codon12/13 | ND | ND | N | MSS |
| Case 66 | 61 | male | right | 4 | LG | present | 4 | dead | 49.5 | ND | ND | ND | N | MSS |
| Case 67 | 71 | male | rectum | 4 | HG | present | 4 | dead | 37.9 | ND | ND | ND | AMP | MSS |
| Case 68 | 60 | male | rectum | 3 | LG | present | 3 | alive | 61.4 | Codon61 | ND | ND | N | MSS |
| Case 69 | 50 | female | left | 4 | LG | present | 4 | dead | 14.4 | Codon12/13 | ND | E545X^†^ | N | MSS |
| Case 70 | 52 | male | rectum | 3 | LG | absent | 2 | alive | 60.9 | Codon12/13 | ND | ND | N | MSS |
| Case 71 | 54 | male | rectum | 3 | LG | present | 4 | alive | 60.5 | ND | ND | ND | N | MSS |
| Case 72 | 43 | female | right | 4 | HG | present | 4 | dead | 18.4 | ND | ND | ND | N | MSS |
| Case 73 | 68 | male | left | 3 | LG | present | 4 | alive | 60.3 | Codon12/13 | ND | ND | N | MSS |
| Case 74 | 60 | male | rectum | 3 | LG | present | 3 | alive | 60.2 | Codon12/13 | ND | ND | N | MSS |
| Case 75 | 52 | male | rectum | 3 | LG | present | 4 | alive | 60.2 | ND | ND | ND | N | MSS |
| Case 76 | 68 | female | left | 4 | LG | present | 4 | alive | 60.1 | Codon12/13 | ND | ND | N | MSS |
| Case 77 | 64 | male | left | 4 | LG | present | 4 | dead | 8.5 | ND | ND | ND | N | MSS |
| Case 78 | 69 | female | right | 3 | LG | present | 4 | alive | 58.5 | ND | ND | ND | N | MSS |
| Case 79 | 71 | male | right | 3 | LG | present | 3 | alive | 58.4 | Codon12/13 | ND | ND | N | MSS |
| Case 80 | 79 | male | left | 3 | LG | present | 3 | alive | 57.5 | ND | ND | ND | N | MSS |
| Case 81 | 77 | female | right | 4 | LG | present | 4 | dead | 4.8 | Codon12/13 | ND | ND | N | MSS |
| Case 82 | 60 | male | rectum | 4 | LG | absent | 4 | alive | 55.9 | ND | ND | ND | N | MSS |
| Case 83 | 51 | male | left | 3 | LG | absent | 4 | alive | 55.1 | ND | ND | E542K | N | MSS |
| Case 84 | 77 | male | left | 3 | LG | present | 4 | dead | 18.2 | Codon61 | ND | ND | N | MSS |
| Case 85 | 47 | female | left | 4 | HG | absent | 4 | alive | 38.4 | Codon12/13 | ND | ND | N | MSS |
| Case 86 | 48 | male | rectum | 3 | LG | present | 4 | alive | 54.9 | Codon12/13 | ND | ND | N | MSS |
| Case 87 | 69 | female | rectum | 3 | LG | present | 3 | alive | 20.6 | Codon12/13 | ND | ND | N | MSS |
| Case 88 | 48 | male | rectum | 3 | LG | present | 4 | alive | 54.4 | ND | ND | ND | N | MSS |
| Case 89 | 56 | female | right | 3 | LG | present | 4 | dead | 23.6 | Codon12/13 | ND | E542K | AMP | MSS |
| Case 90 | 47 | male | left | 4 | LG | present | 3 | alive | 53.8 | Codon12/13 | ND | ND | N | MSS |
| Case 91 | 67 | male | left | 3 | LG | present | 4 | dead | 20.8 | Codon12/13 | ND | E542K | N | MSS |
| Case 92 | 45 | male | right | 3 | LG | present | 3 | alive | 53.1 | Codon12/13 | ND | Q546X^†^ | N | MSS |
| Case 93 | 43 | female | rectum | 4 | LG | present | 3 | alive | 52.4 | Codon12/13 | ND | ND | N | MSS |
| Case 94 | 56 | male | left | 3 | LG | present | 4 | dead | 23.2 | ND | ND | ND | N | MSS |
| Case 95 | 59 | female | rectum | 3 | LG | present | 3 | dead | 37.6 | ND | ND | ND | N | MSS |
| Case 96 | 69 | male | rectum | 4 | LG | present | 4 | dead | 24.4 | ND | ND | ND | AMP | MSS |
| Case 97 | 60 | female | rectum | 4 | LG | present | 4 | dead | 22.9 | Codon12/13 | ND | ND | N | MSS |
| Case 98 | 60 | male | left | 3 | LG | absent | 2 | alive | 51.4 | Codon12/13 | ND | ND | AMP | MSS |
| Case 99 | 62 | female | rectum | 3 | HG | present | 3 | alive | 51 | Codon12/13 | ND | ND | AMP | MSS |
| Case 100 | 60 | female | right | 4 | LG | present | 4 | dead | 27.5 | Codon12/13 | ND | N345K | N | MSS |
| Case 101 | 77 | female | left | 3 | LG | present | 4 | alive | 50.8 | Codon12/13 | ND | ND | N | MSS |
| Case 102 | 65 | female | right | 3 | LG | present | 4 | dead | 39.3 | ND | ND | ND | N | MSS |
| Case 103 | 54 | female | left | 4 | LG | absent | 4 | alive | 50 | Codon12/13 | ND | ND | N | MSS |
| Case 104 | 65 | male | left | 3 | LG | absent | 2 | alive | 49.6 | ND | ND | ND | N | MSS |
| Case 105 | 39 | female | left | 4 | LG | present | 4 | alive | 49.6 | Codon12/13 | ND | ND | N | MSS |
| Case 106 | 68 | male | rectum | 3 | LG | absent | 2 | alive | 49.1 | ND | ND | H1047X^†^ | N | MSS |
| Case 107 | 74 | male | left | 3 | LG | present | 3 | alive | 49.1 | ND | ND | ND | N | MSS |
| Case 108 | 51 | female | rectum | 3 | LG | present | 4 | dead | 0.8 | Codon12/13 | ND | ND | N | MSS |
| Case 109 | 80 | male | right | 3 | LG | present | 4 | alive | 47.9 | Codon12/13 | ND | G1049R | N | MSS |
| Case 110 | 42 | male | rectum | 4 | LG | present | 3 | alive | 47.6 | Codon12/13 | ND | ND | N | MSS |
| Case 111 | 66 | male | right | 3 | HG | present | 4 | dead | 27.5 | Codon12/13 | ND | ND | N | MSS |
| Case 112 | 81 | male | right | 4 | LG | present | 4 | dead | 30.1 | Codon12/13 | ND | ND | N | MSS |
| Case 113 | 34 | female | rectum | 4 | LG | present | 4 | alive | 46.5 | ND | ND | ND | N | MSS |
| Case 114 | 51 | male | rectum | 1 | LG | absent | 4 | alive | 46.2 | Codon12/13 | ND | ND | N | MSS |
| Case 115 | 55 | male | rectum | 3 | HG | present | 4 | alive | 45.8 | ND | ND | R88Q | AMP | MSS |
| Case 116 | 67 | female | left | 3 | LG | present | 3 | alive | 45.8 | Codon12/13 | ND | E545X^†^ | N | MSS |
| Case 117 | 73 | male | rectum | 3 | LG | present | 3 | alive | 45.1 | Codon12/13 | ND | ND | N | MSS |
| Case 118 | 49 | male | right | 3 | LG | present | 3 | alive | 45.1 | Codon12/13 | ND | ND | N | MSS |
| Case 119 | 61 | female | rectum | 4 | LG | present | 4 | dead | 9.5 | Codon12/13 | ND | ND | N | MSS |
| Case 120 | 66 | male | left | 3 | LG | present | 3 | dead | 37.4 | Codon12/13 | ND | ND | N | MSS |
| Case 121 | 82 | male | left | 3 | LG | absent | 2 | alive | 44.2 | ND | ND | ND | N | MSS |
| Case 122 | 65 | male | right | 3 | LG | present | 3 | alive | 44.2 | Codon12/13 | ND | ND | N | MSS |
| Case 123 | 60 | female | right | 4 | LG | present | 4 | dead | 26.8 | ND | V600E | ND | N | MSS |
| Case 124 | 54 | male | left | 3 | LG | present | 4 | dead | 23.6 | ND | ND | E542K | N | MSS |
| Case 125 | 66 | male | right | 3 | LG | present | 3 | alive | 41.6 | Codon12/13 | ND | ND | N | MSS |
| Case 126 | 72 | female | rectum | 4 | LG | present | 4 | alive | 41.4 | ND | ND | ND | N | MSS |
| Case 127 | 59 | male | left | 3 | LG | present | 4 | alive | 41.2 | ND | ND | ND | N | MSS |
| Case 128 | 56 | female | rectum | 4 | LG | present | 4 | dead | 26.4 | ND | ND | ND | AMP | MSS |
| Case 129 | 51 | male | left | 4 | LG | present | 4 | dead | 22 | ND | ND | ND | N | MSS |
| Case 130 | 69 | male | rectum | 3 | LG | present | 4 | dead | 10.9 | Codon12/13 | ND | ND | N | MSS |
| Case 131 | 56 | female | right | 3 | LG | present | 4 | alive | 38.2 | Codon61 | ND | ND | N | MSS |
| Case 132 | 33 | male | left | 3 | HG | present | 4 | alive | 37.9 | ND | ND | ND | N | MSS |
| Case 133 | 73 | male | right | 4 | LG | present | 4 | dead | 7.3 | ND | V600E | ND | N | MSS |
| Case 134 | 56 | female | rectum | 3 | LG | present | 4 | alive | 36.9 | ND | ND | ND | AMP | MSS |
| Case 135 | 60 | male | rectum | 3 | HG | present | 4 | alive | 36.9 | Codon12/13 | ND | ND | N | MSS |
| Case 136 | 51 | female | left | 4 | LG | present | 4 | alive | 36.8 | ND | ND | ND | N | MSS |
| Case 137 | 64 | male | left | 3 | LG | present | 3 | alive | 36.7 | Codon12/13 | ND | ND | N | MSI-H |
| Case 138 | 75 | female | rectum | 2 | LG | present | 4 | dead | 19.4 | Codon12/13 | ND | ND | N | MSS |
| Case 139 | 30 | male | left | 4 | LG | present | 4 | alive | 36.3 | Codon12/13 | ND | ND | AMP | MSS |
| Case 140 | 64 | male | rectum | 3 | LG | present | 4 | alive | 35.8 | ND | ND | ND | N | MSS |
| Case 141 | 68 | female | left | 4 | LG | present | 4 | dead | 10.2 | Codon12/13 | ND | ND | N | MSS |
| Case 142 | 51 | male | rectum | 3 | LG | present | 4 | alive | 34.6 | Codon12/13 | ND | ND | AMP | MSS |
| Case 143 | 71 | female | right | 4 | HG | present | 4 | dead | 4.7 | ND | V600E | ND | N | MSS |
| Case 144 | 43 | male | rectum | 3 | LG | present | 4 | dead | 3.9 | Codon12/13 | ND | ND | N | MSS |
| Case 145 | 35 | female | right | 3 | LG | present | 4 | dead | 23.4 | Codon61 | ND | ND | N | MSS |
| Case 146 | 45 | female | right | 3 | LG | present | 4 | alive | 33.2 | Codon12/13 | ND | ND | N | MSS |
| Case 147 | 77 | male | rectum | 3 | LG | present | 4 | alive | 32.6 | Codon12/13 | ND | E545X^†^ | N | MSS |
| Case 148 | 76 | male | left | 3 | LG | present | 3 | dead | 14.4 | ND | ND | ND | N | MSS |
| Case 149 | 57 | female | rectum | 3 | LG | present | 4 | alive | 32 | ND | ND | ND | N | MSS |
| Case 150 | 64 | female | left | 4 | LG | present | 4 | alive | 31.9 | Codon12/13 | ND | ND | N | MSS |
| Case 151 | 81 | male | left | 4 | LG | present | 4 | alive | 31.8 | Codon12/13 | ND | ND | N | MSS |
| Case 152 | 46 | male | rectum | 4 | LG | present | 3 | dead | 14.6 | ND | V600E | ND | N | MSS |
| Case 153 | 62 | female | left | 4 | HG | present | 4 | alive | 31.5 | Codon12/13 | ND | E545X^†^ | N | MSS |
| Case 154 | 44 | female | rectum | 3 | LG | present | 4 | alive | 31.3 | Codon12/13 | ND | ND | N | MSS |
| Case 155 | 41 | female | left | 3 | LG | present | 4 | dead | 25.8 | ND | ND | ND | N | MSS |
| Case 156 | 55 | male | right | 3 | LG | present | 4 | alive | 31.1 | Codon12/13 | ND | ND | N | MSS |
| Case 157 | 51 | female | right | 3 | LG | present | 4 | alive | 30.9 | ND | ND | ND | AMP | MSS |
| Case 158 | 51 | female | left | 3 | LG | absent | 2 | alive | 30.1 | ND | ND | ND | N | MSS |
| Case 159 | 63 | female | right | 4 | LG | present | 4 | alive | 29.9 | Codon61 | ND | ND | N | MSS |
| Case 160 | 48 | female | right | 4 | LG | present | 3 | alive | 29.8 | ND | ND | ND | N | MSS |
| Case 161 | 64 | female | rectum | 3 | LG | present | 4 | alive | 29.7 | ND | ND | ND | N | MSS |
| Case 162 | 51 | male | left | 3 | HG | present | 4 | dead | 11.8 | ND | ND | ND | N | MSS |
| Case 163 | 69 | male | rectum | 4 | LG | absent | 4 | alive | 29.1 | ND | ND | ND | N | MSS |
| Case 164 | 65 | female | left | 3 | HG | present | 3 | dead | 14.5 | Codon12/13 | ND | ND | N | MSS |
| Case 165 | 61 | female | rectum | 3 | LG | present | 4 | alive | 29 | Codon12/13 | ND | ND | N | MSS |
| Case 166 | 42 | female | rectum | 3 | LG | present | 4 | alive | 28.6 | ND | ND | ND | N | MSS |
| Case 167 | 72 | male | left | 4 | LG | present | 4 | alive | 28.3 | ND | ND | ND | N | MSS |
| Case 168 | 67 | female | left | 3 | LG | present | 4 | alive | 28 | ND | ND | ND | N | MSS |
| Case 169 | 73 | female | right | 4 | LG | present | 4 | alive | 25.7 | ND | ND | ND | N | MSI-H |
| Case 170 | 78 | male | right | 4 | HG | present | 4 | alive | 25.6 | ND | V600E | ND | N | MSS |
| Case 171 | 44 | female | left | 4 | HG | present | 4 | alive | 25.5 | Codon12/13 | ND | ND | N | MSS |
| Case 172 | 67 | male | right | 4 | LG | present | 4 | alive | 25.3 | Codon12/13 | ND | E542K | N | MSS |
| Case 173 | 49 | female | rectum | 4 | LG | absent | 4 | alive | 25.1 | ND | ND | ND | N | MSS |
| Case 174 | 76 | male | rectum | 3 | LG | present | 4 | dead | 16.1 | Codon12/13 | ND | ND | N | MSS |
| Case 175 | 61 | male | left | 3 | LG | present | 4 | alive | 24.6 | ND | ND | ND | N | MSS |
| Case 176 | 65 | female | rectum | 4 | LG | present | 4 | alive | 84.4 | ND | ND | ND | N | MSS |
| Case 177 | 58 | male | left | 3 | LG | present | 4 | dead | 61.7 | ND | ND | ND | N | MSS |
| Case 178 | 66 | male | right | 4 | LG | absent | 4 | dead | 8.5 | Codon12/13 | ND | ND | N | MSS |
| Case 179 | 43 | male | left | 2 | LG | present | 4 | alive | 80.2 | Codon12/13 | V600E | H1047X^†^ | N | MSS |
| Case 180 | 65 | male | left | 3 | LG | absent | 2 | dead | 62.9 | Codon12/13 | ND | E542K | N | MSS |
| Case 181 | 63 | male | left | 4 | LG | present | 4 | dead | 23.9 | Codon12/13 | ND | E542K | N | MSS |
| Case 182 | 75 | male | left | 3 | LG | absent | 2 | alive | 76.2 | ND | ND | ND | N | MSS |
| Case 183 | 70 | male | left | 3 | LG | present | 3 | dead | 69 | ND | ND | ND | N | MSS |
| Case 184 | 59 | male | left | 3 | LG | present | 3 | alive | 75.3 | ND | ND | ND | N | MSS |
| Case 185 | 57 | female | left | 3 | LG | present | 4 | dead | 7 | Codon12/13 | ND | ND | N | MSS |
| Case 186 | 60 | female | left | 3 | LG | present | 3 | alive | 74.8 | Codon12/13 | ND | ND | N | MSS |
| Case 187 | 72 | female | left | 4 | LG | present | 3 | dead | 21 | ND | ND | ND | N | MSS |
| Case 188 | 73 | male | left | 3 | LG | present | 3 | dead | 62.6 | ND | ND | ND | N | MSS |
| Case 189 | 66 | female | rectum | 4 | LG | present | 4 | dead | 26.3 | Codon12/13 | ND | ND | N | MSS |
| Case 190 | 55 | male | rectum | 3 | LG | present | 4 | dead | 37 | ND | ND | ND | AMP | MSS |
| Case 191 | 61 | female | rectum | 3 | LG | present | 4 | dead | 19.3 | ND | ND | ND | AMP | MSS |

LG, low grade (well differentiated, moderately differentiated); HG, high grade (poorly differentiated); FU, follow-up; KRAS, Kirsten rat sarcoma viral oncogene homolog; BRAF, v-raf murine sarcoma viral oncogene homolog B1; ND, not detected; HER2, human epidermal growth factor receptor 2; N, negative; AMP, amplification; MSI, microsatellite instability; MSS, microsatellite stable or MSI-low; MSI-H, MSI-high

*Stage is the stage at initial diagnosis.

^§^Codon12/13 included G112D, G12V, G12C, G12A, G12S, G12R, G12F, G13D, G13C, G13R, G13S, G13A, G13V, and G13I; Codon 61 included Q61H, Q61L, Q61R, Q61K, Q61E, and Q61P of *KRAS* mutation.

^†^H1047X included H1047L, H1047R and H1047Y; Q546X included Q546K, Q546R, Q546E and Q546L; E545X included E545A, E545D, E545G and E545K of *PIK3CA* mutation.
