# Supplementary material for: BRAF, PIK3CA, and HER2 Oncogenic Alterations According to KRAS Mutation Status in Advanced Colorectal Cancers with Distant Metastasis
Source: PLoS One. 2016 Mar 18;11(3):e0151865. doi: 10.1371/journal.pone.0151865 (PMC4798471; doi:10.1371/journal.pone.0151865)
Supplement: S3 Table — (DOCX) [file pone.0151865.s003.docx]

**S3 Table.** Multivariate analysis for factors predictive of survival (Cox proportional hazards model)

| **Factor** | **Hazard ratio (95% CI)** | **P value** |
| --- | --- | --- |
| Age | 1.030 (1.010-1.050) | 0.003 |
| Stage at initial diagnosis (IV vs. Others) | 3.423 (1.957 – 5.986) | <0.001 |
| Mutational status (Others vs. *KRAS*-/*BRAF*+ or *HER2*+) | 2.055 (1.093 – 3.861) | 0.025 |

CI, confidence interval

**Supplementary Table 4.** Association between clinicopathologic characteristics and *BRAF* or *HER2* alterations in *KRAS* wild type CRCs

| Characteristics | Total | *BRAF* or *HER2* alterations | | P value |
| --- | --- | --- | --- | --- |
|  |  | Absent | Present |  |
| Age |  |  |  | 0.966 |
| Mean ± SD | 87 | 59.15 ± 13.13 | 59.00 ± 12.01 |  |
| Sex |  |  |  | 0.645 |
| Male | 48 | 40 (83.3%) | 8 (16.7%) |  |
| Female | 39 | 31 (79.5%) | 8 (20.5%) |  |
| Location |  |  |  | <0.001* |
| Right | 14 | 9 (64.3%) | 5 (35.7%) |  |
| Left | 36 | 36 (100%) | 0 (0%) |  |
| Rectum | 37 | 26 (70.3%) | 11 (29.7%) |  |
| Histologic grade |  |  |  | 0.292* |
| Low | 70 | 59 (84.3%) | 11 (15.7%) |  |
| High | 17 | 12 (70.6%) | 5 (29.4%) |  |
| T stage |  |  |  | 0.095 |
| T1-T3 | 54 | 47 (87.0%) | 7 (13.0%) |  |
| T4 | 33 | 24 (72.7%) | 10 (27.3%) |  |
| pTNM stage† |  |  |  | 0.122* |
| I- | 1 | 1 (100%) | 0 (0%) |  |
| II | 12 | 12 (100%) | 0 (0%) |  |
| III | 15 | 14 (93.3%) | 1 (6.7%) |  |
| IV | 59 | 44 (74.6%) | 14 (25.4%) |  |
| Lymphatic invasion |  |  |  | 0.203 |
| Absent | 28 | 25 (89.3%) | 3 (10.7%) |  |
| Present | 59 | 46 (78.0%) | 13 (22.0%) |  |
| Venous invasion |  |  |  | 0.685 |
| Absent | 56 | 45 (80.4%) | 11 (19.6%) |  |
| Present | 31 | 26 (83.9%) | 5 (16.1%) |  |
| Perineural invasion |  |  |  | 0.050 |
| Absent | 41 | 37 (90.2%) | 4 (9.8%) |  |
| Present | 46 | 34 (73.9%) | 12 (26.1%) |  |

KRAS, Kirsten rat sarcoma viral oncogene homolog; BRAF, v-raf murine sarcoma viral oncogene homolog B1; HER2, human epidermal growth factor receptor 2; SD, standard deviation

Age was compared between two groups by using independent T test.

**P*-values are calculated by using Fisher’s exact test because less than 80% of the cells have an expected frequency of 5 or greater, or any cell has an expected frequency smaller than 1.0.

†Stage is the stage at initial diagnosis.
